# Methylglyoxal hampers the angiogenic capacity of human retinal endothelial cells modifying cell transcriptome with the contribution of NRSF/REST

**DOI:** 10.3389/fcell.2026.1762167

**Published:** 2026-10-02

**Authors:** Marianna Aprile, Cecilia Nigro, Caterina Perfetto, Alessia Leone, Simona Cataldi, Antonella Nicolò, Mariagiovanna Tramontano, Antonella Desiderio, Francesco Beguinot, Valerio Costa, Claudia Miele, Alfredo Ciccodicola

**Affiliations:** 1 Institute of Genetics and Biophysics “Adriano Buzzati Traverso”, National Research Council, Naples, Italy; 2 Institute of Endotypes in Oncology, Metabolism and Immunology, National Research Council, Naples, Italy; 3 Department of Translational Medicine, Federico II University of Naples, Naples, Italy; 4 Department of Science and Technology, University of Naples “Parthenope”, Naples, Italy

**Keywords:** advanced glycation end-product, diabetic retinopathy, dicarbonyl stress, endothelial dysfunction, human retinal endothelial cells, methylglyoxal, NRSF/REST, transcriptional regulation

## Abstract

**Background:**

The functional impairment of retinal microvascular cells is an early and central event in diabetic retinopathy (DR). Methylglyoxal (MGO), the most reactive precursor of advanced glycation end products (AGEs), has been implicated in vascular injury and neuro-retinal dysfunction. Its direct effects on retinal endothelial cells and underlying molecular mechanisms remain poorly understood.

**Methods:**

Human retinal endothelial cells (hRECs) were exposed to MGO and the effect of sublethal doses on the proangiogenic function was evaluated by migration, tube formation and pericyte recruitment assays. MGO derived AGEs were evaluated by western blot. Transcriptome and miRNome profiling were performed to identify gene expression and microRNA perturbations. Integrative *in silico* analyses combining RNA-Seq with ENCODE ChIP-Seq datasets were used to identify transcriptional regulators responsible for MGO-induced gene deregulation. Transcriptomic profiles were compared with independent human DR datasets, and *REST* was silenced by siRNAs followed by analyses of proangiogenic function.

**Results:**

The exposure of hRECs to MGO for 72 h is sufficient to increase protein glycation adducts, impair the migration, tube-formation and the recruitment of pericytes. Transcriptome and miRNome analyses highlight that MGO induces extensive transcriptional perturbations, related to gene expression regulation, cell cycle, cell death and vasculature development. Among the identified transcriptional regulators, Neuron-Restrictive Silencer Factor/RE1-Silencing Transcription Factor (NRSF/REST) emerges as a candidate mediator, with binding sites enriched in 56% of deregulated genes. NRSF/REST expression markedly increases after 24 h of MGO treatment, suggesting it is an early event of the exposure to MGO. Protein–protein interaction network analysis prioritized NRSF/REST as a central node linking key MGO-responsive pathways. Accordingly, NRSF/REST knockdown is associated with the reduction of *TP53* and *FOSL2*, which are induced by MGO and display NRSF binding sites and known migration-related functions. Importantly, *REST* silencing significantly attenuates MGO-induced defects in endothelial migration, tube formation and pericyte recruitment, supporting its contribution to both transcriptional and functional endothelial alterations induced by MGO in hRECs.

**Conclusion:**

These findings demonstrate that MGO exposure disrupts endothelial function and transcriptional homeostasis in hRECs, identifying NRSF/REST as a contributor to MGO-induced endothelial dysfunction and supporting further investigations of the MGO/NRSF-REST axis as a potential therapeutic target for DR.

## 1 Introduction

Diabetic retinopathy (DR) is a leading cause of blindness and visual impairment, occurring with variable severity in all patients with type 1 diabetes (T1DM) and about 60% type 2 diabetic (T2DM) patients (Wong et al., 2016; Hietala et al., 2010). Genetic factors contributing to DR onset or susceptibility have been reported, but the heritability of DR is still a matter of debate (Hietala et al., 2010; Arar et al., 2008; Costa and Ciccodicola, 2012; Li et al., 2023).

One of the primary events occurring in the retina of diabetic individuals consists of the functional impairment of microvascular cells (i.e., pericytes and endothelial cells) and other cell types constituting the neurovascular unit (e.g., Muller and glial cells) (Aiello et al., 1998; Hammes, 2018; Saxena et al., 2025). This damage is induced by chronic hyperglycemia, and the accumulation of glucose-metabolites further increases retinal damage’s susceptibility. Moreover, cellular damage further promotes the inflammatory and oxidative environment, which in turn sustains the dysfunctional crosstalk in the neurovascular unit perpetuating the vascular damage and the unproductive angiogenesis (Brucklacher et al., 2008; Günthner et al., 2019; Capitão and Soares, 2016; Tang et al., 2024).

In DR, similar to other diabetic complications, the accumulation of advanced glycation end products (AGEs) is increasingly recognized as a pivotal factor contributing to cellular dysfunctions and particularly to the damage of microvascular cells (Baynes, 1991; Cui et al., 2006; Kang and Yang, 2020; Toma et al., 2026; Oshitari, 2023; Portillo et al., 2024). The dicarbonyl methylglyoxal (MGO) is the most reactive AGE precursor and conditions favoring its accumulation, such as the increased glycolitic flux due to chronic hyperglycemia, cause dicarbonyl stress. This means that its excessive accumulation in plasma and tissues leads to AGEs formation by MGO irreversible reaction mainly with arginine residues. Dicarbonyl stress favors cell apoptosis, inflammation and endothelium dysfunction (Brouwers et al., 2004; Yao et al., 2007; Mukohda et al., 2009; Queisser et al., 2010; Mukohda et al., 2013; Allaman et al., 2015; Vulesevic et al., 2016; Sachdeva et al., 2018; Prevenzano et al., 2022; Liu et al., 2023; Klochkov et al., 2024; Wen et al., 2026). Notably, in absence of hyperglycemia, MGO exogenous administration is sufficient to induce neuroretinal dysfunction, vascular damage and retinal lesions (Schlotterer et al., 2019). Indeed, the glycation of protein residues induced by MGO can, *per se*, affect the modulation of gene expression, including the specific alteration of transcriptional modulators and non-coding RNAs (ncRNAs) (Yao et al., 2007; Ramasamy et al., 2006; Schalkwijk and Stehouwer, 2020). Accordingly, the exposure to high MGO levels affects the expression of Ang-2, BAX, Bcl-2 (Yao et al., 2007; Mukohda et al., 2009; Queisser et al., 2010; Mukohda et al., 2013; Allaman et al., 2015; Vulesevic et al., 2016; Sachdeva et al., 2018; Prevenzano et al., 2022; Liu et al., 2023; Klochkov et al., 2024; Wen et al., 2026; Schlotterer et al., 2019; Ramasamy et al., 2006; Schalkwijk and Stehouwer, 2020; Bento et al., 2010) and different microRNAs (miRNAs) in endothelial cells (Desai et al., 2010; Nigro et al., 2017). Therefore, a comprehensive investigation of the functional and molecular effects of MGO on retinal endothelial cells represents a crucial step for advancing our understanding of DR pathogenesis, fostering the development of novel therapeutic strategies (Lin et al., 2025). Indeed, although complex interactions among endothelial cells, pericytes, Müller glia, neurons, immune cells and the extracellular matrix occur within the retinal neurovascular unit, reductionist *in vitro* models remain essential to dissect cell-autonomous molecular mechanisms that are otherwise difficult to resolve *in vivo.* In this context, primary human retinal endothelial cells (hRECs) provide a suitable platform to investigate the direct endothelial response to MGO independently of systemic diabetic cues.

Here, we investigated the functional and molecular effects induced by sublethal doses of MGO in hRECs by combining integrated transcriptome- and miRNome-wide analysis with computational validation in independent DR datasets. Our findings reveal that MGO affects endothelial homeostasis–in terms of cell migration, tube formation, and pericyte recruitment - inducing a wide reshaping of cell transcriptome and miRNome. Notably, we identify the Neuron-Restrictive Silencer Factor/RE1-Silencing Transcription factor (NRSF/REST) as a potential mediator of the transcriptional response to MGO. The observation that MGO upregulates NRSF/REST, whose silencing counteracts the MGO-induced defects in migration, tube formation, and pericyte recruitment, supports the contribution of NRSF/REST to endothelial dysfunction and identifies the MGO/NRSF-REST as a promising therapeutic axis for DR.

## 2 Materials and methods

### 2.1 Cell cultures and treatments

Primary hRECs, purchased by Innoprot (Derio, Bizkaia, Spain; REF: P10880), are isolated from human healthy retina from a single donor, cryopreserved at passage one, delivered frozen, and guaranteed to further expand for 10 population doublings. Also, hRECs are characterized for vWF/Factor VIII, CD31 (P-CAM) and uptake of Dil-AC-LDL. Cells were cultured in optimized medium and reagents (Innoprot) for their growth; i.e., Endothelial cell medium (ECM) supplemented with fetal Bovine Serum (FBS), Endothelial Cell Growth Supplement (ECGS) and penicillin/streptomycin (P/S) solution, which are quality tested together and guaranteed to give maximum performance as a global solution for *in vitro* hREC culture. Experiments were repeated using independent vials from different cell batches at low passage number. Therefore, biological reproducibility was assessed across independent hREC preparations, although donor-to-donor variability could not be evaluated in this study. Human Retinal Pericytes (hPCs), purchased by Innoprot (Derio, Bizkaia, Spain; REF: P10869), were cultured in Pericytes medium supplemented with FBS, Pericyte Growth Supplement (PGS) and P/S solution, according to the instructions provided in the technical sheet (ECM; Innoprot). Cell adhesion and spreading were supported by treatment of culture surfaces with bovine plasma fibronectin or poly-L-lysine (Innoprot) for hRECs and hPCs, respectively. Dose-response and time-course experiments were performed by treating cells with MGO (Sigma-Aldrich, St Luis, MO, United States), as specified in figures and legends. According to viability assays, the treatment with 500 μM MGO for 72 h was selected as non-toxic and chronic exposure for analysis of migration, tube formation, cell transcriptome and miRNome analysis. Targeted gene expression analysis was also assessed in hRECs exposed to 500 μM MGO or vehicle (Veh) starting at the following 24 h.

### 2.2 Cell transfection

Two different *REST* siRNAs (i.e., siRNA1 and siRNA2) were purchased from Integrated DNA Technologies (IDT, Coralville, Iowa, United States) and tested by scaling siRNA concentrations. Cells were transfected by Lipofectamine3000 (Life Technologies, Carlsbad, CA, United States) in culture medium without antibiotics and serum, then replaced after 6 h with complete culture medium. Silencing efficacy was assessed by qPCR using cells transfected with scrambled siRNAs as reference samples. According to the results obtained, for further assays hRECs were transfected with *REST* siRNA2 (25 nM) for 48 h.

### 2.3 RNA extraction, RT-PCR and qPCR

RNA samples were extracted by cell pellets using TRIzol Reagent (Life Technologies, Carlsbad, CA, United States) according to the manufacturer’s instructions. NanoDrop spectrophotometer (Life Technologies, Carlsbad, CA, United States) was used for RNA quantification and cDNA samples were obtained by High-Capacity cDNA Reverse Transcription kit (Invitrogen, Carlsbad, CA, United States), as indicated on datasheet instructions. CFX Connect Detection System (Bio-Rad, Hercules, CA, United States) and iTaq Universal Sybr Green Supermix (Bio-Rad, Hercules, CA, United States) were used for qPCR assays, according to manufacturer’s instructions. Specific primer pairs for qPCR assays were designed by Oligo 4.0. software (Supplementary Table S1) and tested by RT-PCR using AmpliTaq Gold (Life Technologies, Carlsbad, CA, United States), agarose gel electrophoresis and Sanger sequencing. For evaluating the relative mRNA abundance, expression analysis was performed by CFX Manager™ Software by 2^−ΔΔCT^ method and setting to 1 the expression values in control cells (i.e., the reference sample). The expression stability of different housekeeping genes (i.e., *PPIA, HPRT, GAPDH* and *RPS23*) was evaluated and PPIA was chosen as the reference gene. All reactions were performed at least in triplicate.

### 2.4 Tube formation assay

The tube formation was assessed on growth factor-reduced Matrigel (BD Biosciences) as described by Liu et al. (Liu et al., 2012). Briefly, after treatment, hRECs were starved for 3 h. Then, 1 × 10^4^ cells/well were seeded on Matrigel-coated 96-well plates and incubated at 37 °C for 6 h. Tube formation was analyzed by measuring the number of tubes and nodes and quantified using ImageJ software. Images were acquired from randomly selected fields within predefined regions of each well. Image quantification was performed by an investigator blinded to the treatment groups, using identical analysis parameters across all experimental conditions.

### 2.5 Migration assay

Cells were starved without serum for 5 h. After trypsinization, 4 × 10^4^ cells/well were plated on the upper side of 12-well cell culture inserts (BD Falcon, Franklin Lakes, NJ) with 8 µm pores polyethylene terephthalate (PET) for evaluating the migration of hRECs treated or not with MGO. Culture medium with or without 10% FBS was added to the bottom of the well, as chemotactic stimulus. In co-culture experiments, 2.5 × 10^4^ hRECs/well were first plated on a 24-well plate, transfected with siRNA and treated or not with MGO, where indicated. Then, 5 hours-starved hPC (2.5 × 10^4^/insert) were plated on the upper side of 24-well cell culture inserts for evaluating the ability of hRECs to induce hPCs recruitment. In both cases, following 24-h incubation, the top of the insert was cleared of cells with a cotton swab. The top and bottom of the insert were washed 3 times with 1× PBS. Migrated cells were fixed with glutaraldehyde, stained with Crystal Violet, and counted as previously reported (Nigro et al., 2019). Images were acquired from randomly selected fields within predefined regions of each well. Image quantification was performed by an investigator blinded to the treatment groups, using identical analysis parameters across all experimental conditions.

### 2.6 Western blot

Total protein lysates were obtained and separated by SDS-PAGE as previously described (Nigro et al., 2019). In detail, hRECs were solubilized in lysis buffer (50 mmol/L HEPES, pH 7.5, 150 mmol/L NaCl, 10 mmol/L EDTA, 10 mmol/L Na2P2O7, 2 mmol/L Na3VO4, 100 mmol/L NaF, 10% glycerol, 1% Triton X-100, 1 mmol/L phenylmethylsulfonylfluoride and 10 μg/mL aprotinin) for 2 h at 4 °C. Cell lysates were clarified by centrifugation at 16.000 g for 20 min. To analyze the accumulation of MGO-adducts, 40 μg of protein lysates were loaded and separated by SDS-PAGE and then transferred onto 0.45 μm Immobilon-P membranes (Millipore). Accumulation of MGO-adducts was detected by incubating membranes with the anti-MGO antibody (Abcam, Cambridge, UK). For normalization, anti-Vinculin antibody (Santa Cruz Biotecnology, CA, United States) was used. Immmunoreactive bands were detected by chemiluminescence according to the manufacturer’s instructions (Pierce, Thermo Scientific, Rockford, IL, United States). Densitometric analysis was performed using ImageJ software.

### 2.7 Statistical analysis

All non-omics experimental assays (viability, tube formation, migration, western blot, and qPCR) were performed at least in triplicate and results are shown as means of different experiments with standard error of the mean (SEM), as described in figure legends. For viability, tube formation, migration, western blot and qPCR assays, comparison between groups were performed using Student’s t-test and p ≤ 0.05 was considered statistically significant. For assessing gene expression variations values of control samples were set at 1 (one sample test; two tailed) and normal data distribution was verified by the Shapiro–Wilk test (“shapiro.test function”) implemented in R language. Statistical analyses for transcriptomic, smallRNA-Seq, and publicly available datasets follow dedicated pipelines described in the sections below, including method-specific normalization, differential expression testing, and multiple testing correction strategies.

### 2.8 Transcriptome and miRNome data generation and analysis

Total RNA samples of hRECs exposed to MGO (500 μM MGO for 72 h) or Veh (CTR) (n = 6) were quantified by Qubit (Life Technologies, Carlsbad, CA, United States). RNA-Seq and small-RNASeq analysis were performed by IGA Technology Services (Udine, Italy). Transcriptome and smallRNA-Seq raw data were analyzed following previously published pipelines (Costa et al., 2017; Aprile et al., 2018), with modifications described below.

#### 2.8.1 RNA-Seq data processing and differential gene expression analysis

Transcriptome analysis was conducted on poly-A + RNA fraction (with 3 biological replicates per group), by generating paired-end reads (2 × 150 bp) and using NovaSeq6000 platform (Illumina). Read quality was assessed with FastQC (version 0.11.9) and reads with adapter contamination or Phred score <20 were trimmed/excluded from further analytical steps. Reads were then aligned to the human reference genome (hg19) and GENCODE v19 transcriptome-guided alignment was carried out using TopHat (version 2.1.1) (Trapnell et al., 2012) with specific parameters: 2 read mismatches (-N 2), 10 mismatches in the segment gap region (-g 10), mate inner distance 200 with 15bp standard deviation, 1 alignment reported per read (-m 1), fr-secondstrand library type, 3 segment mismatches, and a 2bp read edit distance. Gene-level read counts were generated and processed using RNASeqGUI (Russo and Angelini, 2014). Low-count genes were filtered (CPM>1 in at least one sample), retaining about 13,300/57,820 genes (23.0%); filtered counts were normalized using the upper-quartile method (UQUA; Bullard et al., 2010). Gene quantification was carried out using *FeatureCounts* option - implemented in RNAseqGUI. Resulting data have been inspected using Principal Component Analysis and Scatter Plot Matrix functions.

Differential expressions were tested in EdgeR fitting a negative binomial generalized linear model (GLM) with dispersion estimated by the exact test. Genes were considered significantly differentially expressed at a false discovery rate (FDR) < 0.05 (Benjamini–Hochberg correction) (Tarazona et al., 2011) combined with an absolute log2 fold-change threshold of |log2FC|>1, yielding 1802 differentially expressed genes.

For the analysis of biological processes, molecular functions, and pathways of DEGs, the following databases and tools were used: KEGG (Kanehisa and Goto, 2000; Kanehisa et al., 2023); DAVID Gene Functional Classification Tool (Huang et al., 2007), PANTHER Classification System (Thomas et al., 2003; Mi et al., 2013), basing prioritization on adjusted enrichment significance. To define predictive network-based associations, a hypothesis-generating framework to prioritize main candidate regulators of DEGs was generated by the analysis of Protein-Protein Interaction (PPI) network using the STRING tool (Interaction evidence; Confidence of score: high>0.700) (Szklarczyk et al., 2011) and ENCODE ChIP-Seq Significance Tool (hypergeometric Test; Benjamini Hochberg; Q-value<0.05) (Auerbach et al., 2013).

#### 2.8.2 SmallRNA-seq processing and miRNA differential expression analysis

Moreover, smallRNA-Seq was conducted on the NextSeq500 platform (Illumina) by generating single reads (1 × 75bp). Raw reads were processed using iSmaRT v 1.0 (Panero et al., 2017), which integrates Cutadapt for adapter removal and sRNAbench as its core engine for read mapping, profiling, and normalization. Adapter trimming was performed with Cutadapt using default parameters (minimum read length 17 nt, error rate 0.1, quality cutoff 0), followed by sRNAbench pre-processing (minimum read length 15 nt, maximum read length 50 nt, one mismatch allowed in adapter detection). Reads were mapped to the human reference genome (hg19) and miRBase mature/hairpin sequence libraries using sRNAbench default profiling parameters (zero mismatches allowed against mature and hairpin sequences). RPM normalization was applied given the short-read length and mature short-readsed profiling strategy. NOISeq was selected due to its non-parametric framework suitable for small RNA libraries with moderate dispersion characteristics. Differentially expressed miRNAs were identified applying a probability threshold of 0.90, a value selected to provide intermediate stringency between the conventionally used 0.80 (4:1 odds) and the more conservative 0.95 threshold recommended for biologically replicated designs.

The miRNA Pathway Dictionary Database (miRPathDB release 2.0) (Kehl et al., 2020) was used to identify significantly enriched pathways and cellular components targeted by differentially expressed miRNAs. Heatmap Calculator tool was adopted considering only interactions supported by strong experimental evidence. Pathway enrichment was based on the Reactome database, selecting pathways targeted by at least three miRNAs and including a minimum of ten deregulated miRNAs. Cellular component enrichment was performed using the Gene Ontology annotation, applying the same filtering criteria (cellular components targeted by at least three miRNAs and including at least ten deregulated miRNAs). miRNA–mRNA interaction networks were generated using miRNet (Chang et al., 2020), based on miRTarBase v4 interactions and miRBase release 22 (June 2019) as the miRNA annotation source.

#### 2.8.3 Re-analysis of publicly available transcriptomic datasets

For translational validation in public datasets, raw RNA-seq counts were obtained from GEO (GSE94019, GSE228900) (Lam et al., 2017; Maurissen et al., 2024) and processed with edgeR (TMM normalisation, voom-limma). For GSE228900 — a transcriptomic dataset of primary human retinal microvascular cells in a diabetic inner blood-retinal-barrier-on-a-chip (iBRB) (Maurissen et al., 2024) — diabetic (Cond3) versus untreated control (Cond1), microvascular networks at matched timepoints were compared. For GSE94019 — laser-captured retinal vascular endothelial cells from patients with proliferative diabetic retinopathy (PDR) (Lam et al., 2017) — PDR fibrovascular membranes were compared to non-diabetic retina. GSEA was performed with fgsea using MGO-upregulated and -downregulated gene signatures (FDR<0.05). Concordance was assessed by rank-rank logFC correlation and directional agreement.

## 3 Results

### 3.1 MGO exposure impairs the angiogenic ability of hRECs

To investigate the intracellular effects of MGO in human retinal microvascular endothelial cells, we employed commercially available primary hRECs as the experimental model. To find the optimal experimental condition for our investigations, we exposed hRECs to different doses of MGO (Figure 1A) from 12 to 72 h (Figure 1B). MTT assay shows that chronic exposure (72 h) to up to 500 μM MGO does not impair cell viability of hRECs. The same condition is enough to increase the intracellular accumulation of MGO-modified proteins by 70%, as compared to not-treated control cells (Figure 1C). The functional effect of MGO exposure was investigated by testing some crucial aspects of the angiogenic process (i.e., cell migration, tubular structure formation and microvessel stabilization), in which endothelial cells are the main actors. To this aim transwell assays were performed showing that MGO treatment reduces by 30% the hREC migration in response to FBS (10%), used as pro-angiogenic stimulus, compared to not treated control cells (Figure 1D). Furthermore, the ability to form tubular structures was evaluated in hRECs by testing the number of nodes and loops formed in an extracellular matrix extract, which are both reduced by 70% in MGO-treated compared with control cells (Figure 1E). Primary hPCs and hRECs were plated in a co-colture system to test the migration of hPCs toward hRECs, as a functional effect of cellular crosstalk, which is essential for physiological microvessel stabilization. Obtained data show that the ability to recruit hPCs is reduced by 30% in MGO-treated hRECs (Figure 1F). These results demonstrate that hRECs exposed to sublethal doses of MGO and accumulating MGO-modified proteins, characteristic of a dicarbonyl stress condition related to diabetes mellitus, have an impaired angiogenesis ability.

**FIGURE 1:**
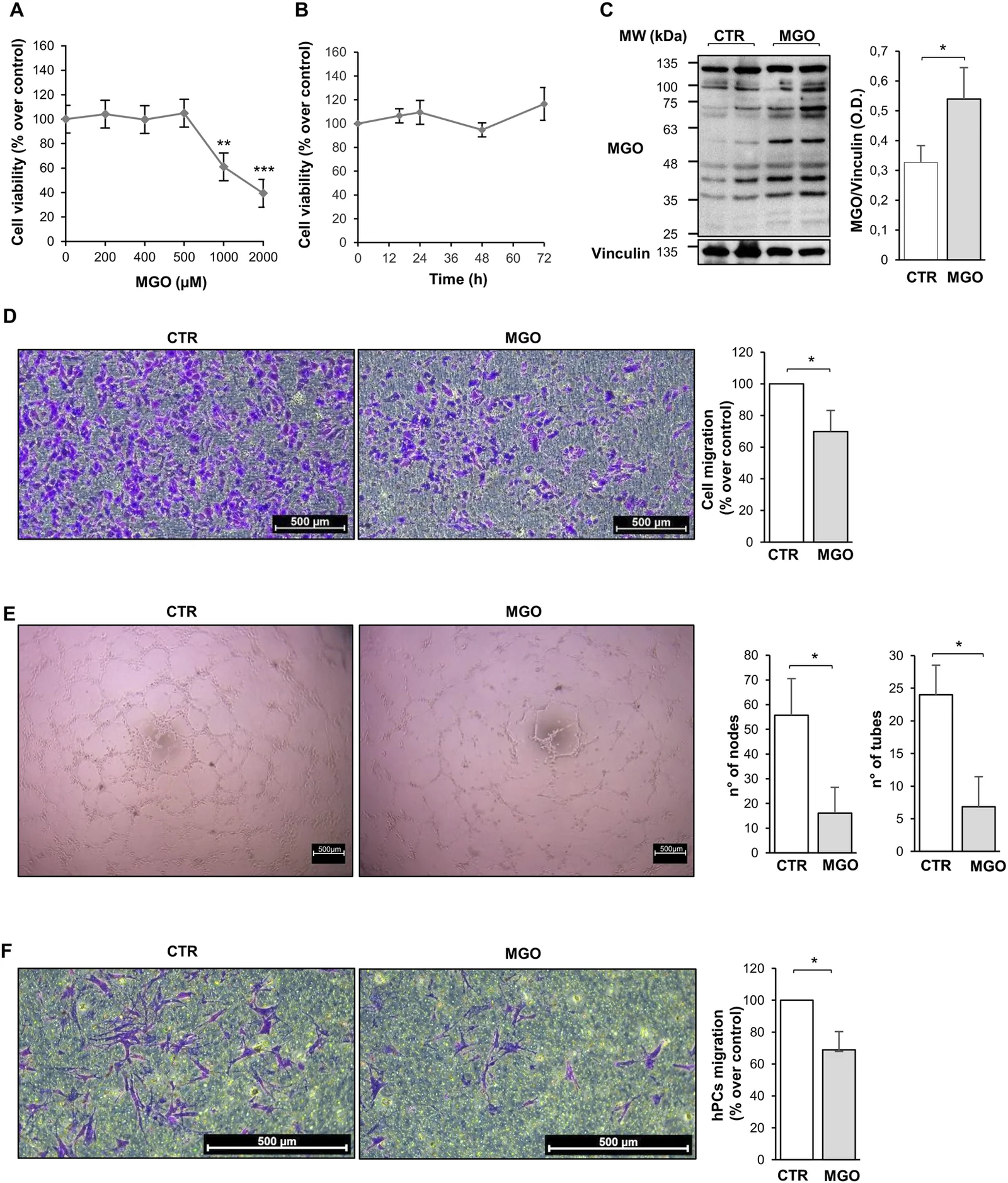
Methylglyoxal induces endothelial dysfunction in hRECs. **(A,B)** Relative cell viability (%) in hRECs upon treatment with increasing doses of MGO **(A)** and with MGO 500 μM (selected as not-toxic concentration) over 72 h **(B)**. Data are reported as mean ± SEM vs. untreated control cells (CTR, set to 100% of viability) in independent experiments [n = 6 in **(A)**; n = 4 in **(B)**]. Comparison between groups were performed using Student’s t-test, with **p ≤ 0.01 and ***p ≤ 0.001. **(C)** Representative autoradiographs of Western blot analysis of MGO-protein adducts (left panel) and densitometry analysis (right panel) in hRECs treated or not (CTR) with MGO 500 μM for 72 h (MGO). Multiple bands *per* lane (left panel) show a spectrum of proteins of different molecular weight that are modified by MGO. MGO-modified protein levels were quantified by the densitometric analysis of each sample lane and normalized on the vinculin specific band, used as a loading control. Densitometry data are reported as mean ± SEM of independent experiments (n = 8). Statistical comparison between groups was performed using Student’s t-test, with *p ≤ 0.05. **(D)** Representative optical microscopy images at ×5 magnification (scale bar: 500 μm) of migrated hRECs treated or not with MGO, exposed to FBS as pro-angiogenic stimulus and stained with crystal violet after 24 h. Bar graph indicates the mean ± SEM of the number of migrated hRECs in independent experiments (n = 6), expressed as % over control. Comparison between groups was performed using Student’s t-test, with *p ≤ 0.05. **(E)** Representative optical microscopy images at ×4 magnification (scale bar: 500 μm) of hRECs treated or not with MGO, seeded on Matrigel-coated 96-well plate and analyzed after 6 h. Bar graphs indicate the number of nodes (left panel) and tubes (right panel), analyzed and quantified by ImageJ. Data are reported as mean ± SEM of independent experiments (n = 3). Comparison between groups was performed using Student’s t-test, with *p ≤ 0.05. **(F)** Representative optical microscopy images at ×10 magnification (scale bar: 500 μm) of migrated hPCs in response to co-culture with hRECs treated (right image) or not (left image) with MGO, and stained with crystal violet after 24 h. Bar graph indicates the mean ± SEM of the number of migrated hPCs in independent experiments (n = 6), expressed as % over control. Comparison between groups was performed using Student’s t-test, with *p ≤ 0.05.

### 3.2 MGO reshapes the transcriptome of hRECs

Thus, considering these major phenotypic changes impacting the functionality of endothelial cells, we sought to investigate the impact of MGO on the transcriptional program of hRECs. To this aim, we combined standard polyA + mRNA-Seq and smallRNA-Seq to profile the transcriptome and miRNome, respectively, of MGO-exposed vs. control hRECs (Supplementary Figure S1A). The high-throughput of RNA-Seq analysis was confirmed by the capacity of this approach to simultaneously measure the expression of about 13,300 genes (Supplementary Material 1). Interestingly, the expression of about 5,800 genes - out of the ≈12,200 genes with a known function in DAVID database (i.e., ≈47%) - is enriched in the human brain (Supplementary Material 1), supporting the “neuron-like” signature of the hRECs. This finding, in line with the peculiar role of the microvascular retinal endothelial cells in the neurovascular unit, further confirmed the *bona fide* of this *in vitro* model, prompting us to further explore the MGO-induced molecular perturbations in these cells.

To systematically rule out the effects of MGO on the transcriptional network of hRECs, we focused on differentially expressed genes (DEGs; ≈1800) between MGO-exposed and control hRECs (Figure 2A; Supplementary Figure S1B, Supplementary Material 1). Notably, more than 20% of these genes (i.e., 371 genes) are classified as DR-related in the GeneCards Human Gene Database (Supplementary Material 1), suggesting a relevant overlap with disease-associated transcriptional programs. Accordingly, gene set enrichment analysis showed that genes deregulated by MGO in hRECs are significantly and concordantly enriched in diabetic primary human retinal microvascular cells (GSE228900, (Kanehisa et al., 2023); Supplementary Figure S1C).

**FIGURE 2:**
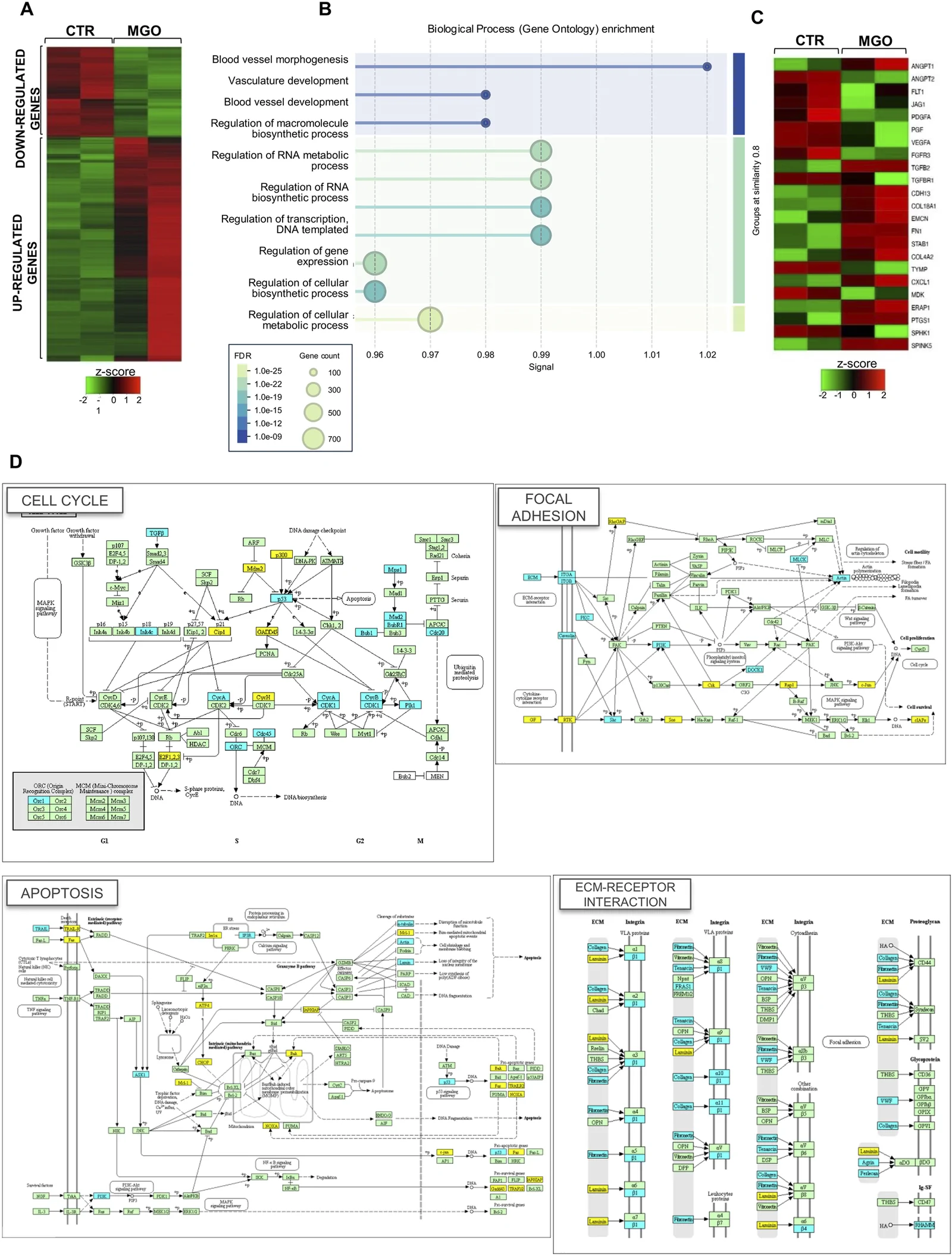
MGO induces transcriptional changes and affects angiogenesis-related pathways. **(A)** Heatmap generated in R displaying normalized expression data of differentially expressed genes (DEGs) analyzed by RNA-Seq analysis in hRECs treated with MGO 500 μM for 72 h (MGO) and hRECs treated with Veh (CTR). **(B)** Gene ontology analysis by STRING showing the main biological pathways enriched of DEGs, relative gene counts and FDR. **(C)** Heatmap generated in R displaying normalized expression data of DEGs - between hRECs treated with MGO and Veh (CTR) - involved in angiogenesis. **(D)** Graphical representations - generated using KEGG database - of selected pathways enriched of genes differentially expressed between hRECs exposed to MGO and CTR. Blue and yellow squares indicate proteins/complexes encoded by downregulated and upregulated genes, respectively.

Gene Ontology and Pathway analysis - performed on the 1,283 up- and 519 downregulated genes (Supplementary Material 1) - revealed that MGO exposure has wide and heterogeneous effects on multiple molecular functions (Figure 2B), in line with its capacity to extensively form glycation adducts on DNA, RNA and proteins. Nonetheless, we identified a marked enrichment in specific cellular pathways, such as “apoptosis” and “p53-,” “integrin-” and “gonadotropin-releasing hormone receptor signaling” (Supplementary Figure S1D). We also observed that most of the genes deregulated upon MGO exposure are involved in cell death, migration and adhesion, cell cycle, proliferation, vasculature development and largely in gene expression regulation (Figure 2B; Supplementary Figure S1E). Specifically, in line with their impaired functionality, MGO-exposed hRECs exhibit altered expression of key genes involved in angiogenesis (Figure 2C). A significant fraction of the altered genes belonging to the above-mentioned biological processes takes part in the transcriptional regulation, further confirming that MGO may exert its main detrimental effects through a deregulation of gene transcription (Supplementary Figures S1D–F). Transcriptome findings suggest that the alteration of genes/proteins involved in the regulation of gene expression may represent an early event, which triggers the perturbation of cellular pathways associated with motility, angiogenic capacity and other DR-related molecular dysfunctions (e.g., adhesion, cell cycle, proliferation, vasculature development, Figure 2D).

Interestingly, small-RNA-Seq analysis (Supplementary Figure S1A) further confirmed the biological processes and pathways that are perturbed by MGO. Indeed, 83 miRNAs were identified as differentially expressed upon MGO exposure (Figure 3A, Supplementary Material 2) and the analysis of the targeted protein-coding genes (i.e., displaying high-confident binding sites for differentially expressed miRNAs; Supplementary Material 2) revealed prevalent nuclear localization and strong enrichment for pathways and biological processes related to the transcriptional regulation (Figure 3B; Supplementary Figures S2A,B). This finding further corroborates the impact of MGO on gene expression regulation. Notably, combining transcriptome and miRNome data, we identified a core subset of MGO-altered mRNAs (i.e., 718/1803; ≈40%) that are targeted by MGO-altered miRNAs (i.e., 68 miRNAs; target score≥85; Figure 3C; Supplementary Material 2). Our integrated analysis also revealed that about half of the core subset of MGO-perturbed genes (i.e., 329/718; ≈46%; Supplementary Material 2) is targeted by seven miRNAs (i.e., miR-186-5p, miR-92a-3p, miR-26b-5p, let-7b-5p, miR-155-5p, miR-16-5p, hsa-miR-23a-3p) that are deregulated upon MGO exposure. Noteworthy, these mRNA-miRNA pairs belong to the above-discussed pathways, including gene expression regulation, cell proliferation/differentiation and motility. Therefore, our analysis - based on the combination of two complementary omics approaches - revealed that MGO produces ample transcriptional deregulation in hRECs, suggesting that the functional alteration of regulators of the gene expression may represent - at least - one of the primary intracellular events underlying the phenotypic and functional deficit of microvascular retinal cells.

**FIGURE 3:**
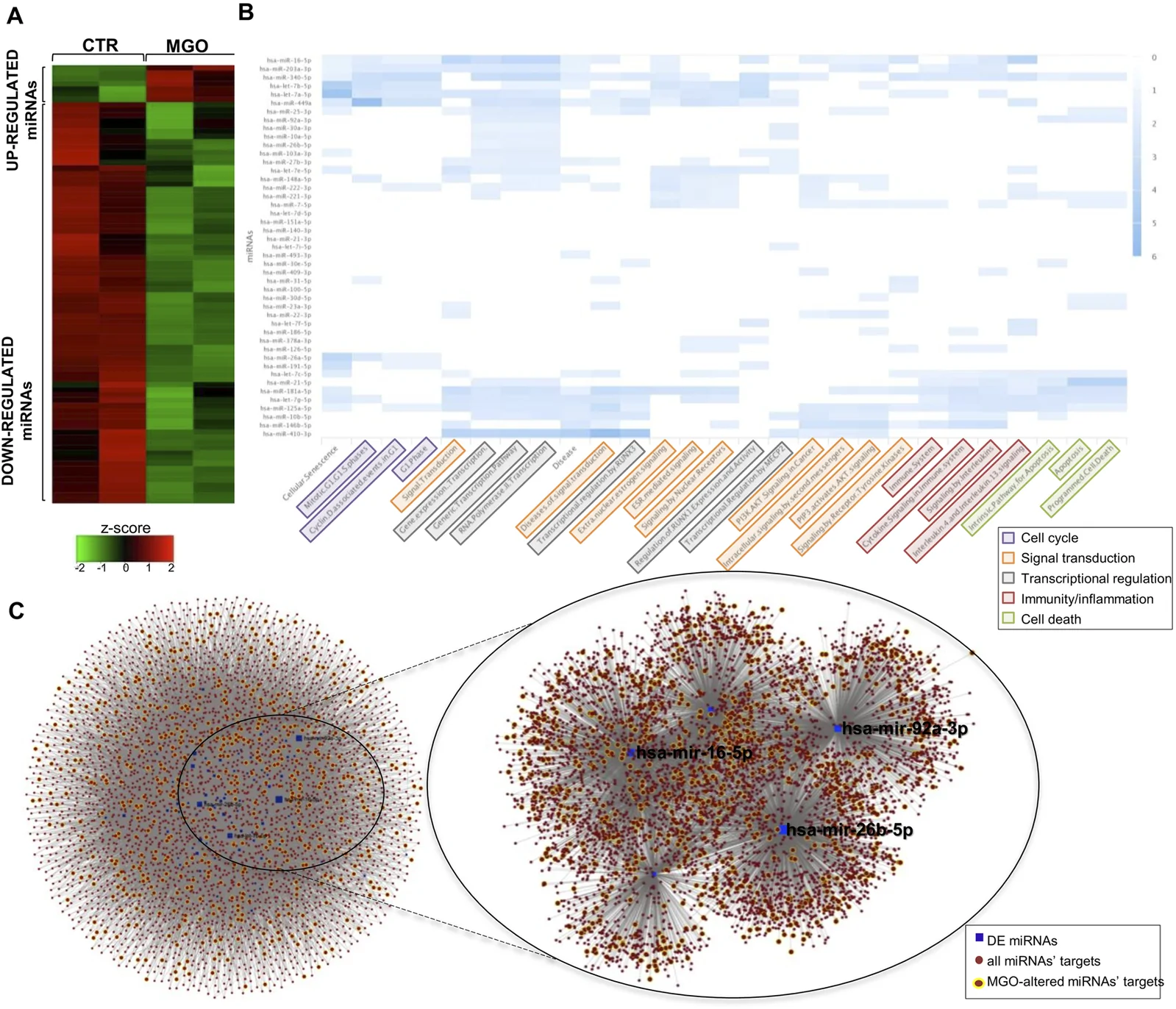
MGO alters miRNome and miRNA-mediated regulatory networks. **(A)** Heatmap generated in R displaying normalized expression data of differentially expressed miRNAs analyzed by smallRNA-Seq analysis in hRECs treated with MGO 500 μM for 72 h (MGO) and hRECs treated with Veh (CTR). **(B)** Heatmap generated by miRPathDB 2.0 of biological processes of genes targeted by deregulated miRNAs, according to the KEGG database. Different colors indicate the main biological processes including the specific signaling/processes. **(C)** Network generated by miRnet tool of deregulated miRNAs (blue squares) representing the nodes and targeted mRNAs (red circles) including MGO-altered genes (DEGs; red circles with yellow borders) (left panel), with a zooming of the network of five selected miRNAs (right panel).

### 3.3 NRSF/REST is unveiled as central hub in the transcriptional network perturbed by MGO in hRECs

MGO exposure exerts significant effects on the expression of genes accounting for transcriptional regulation and related pathways. Therefore, we sought to identify which transcriptional regulator/s may be responsible for the transcriptome-wide effects observed in hRECs upon MGO exposure. Therefore, integrating RNA-Seq datasets and ENCODE ChIP-Seq data, we selected all the transcriptional regulators (altered upon MGO exposure) whose binding sites (ChiP-Seq peaks) are significantly enriched in the promoters of genes altered upon MGO exposure (Supplementary Material 3). The top ranked factors (Figure 4A) were selected as candidate factors mediating the MGO-induced phenotypic and functional effects. Basic/general transcription factors displaying very low expression levels in hRECs (in RNA-Seq data sets) and those showing target genes with a large overlap for other factors were excluded (Figures 4A,B). Using these criteria, the analysis revealed the NRSF/REST displaying the highest number of targeted DEGs and of specific targets (Figures 4A,B), making NRSF/REST a strong candidate for further analyses. Supporting this prioritization, *REST* is highly expressed in hRECs and shows the most significant enrichment value for the presence of binding sites (ChIP-Seq peaks; Q ≤ 0.05), in more than half of the DEGs (i.e., 1009/1803, ≈56%; Figure 4B, Supplementary Material 3). Interestingly, focusing on the subset of genes whose promoter is bound by NRSF/REST, we found a significant enrichment in “transcriptional regulation” (i.e., ≈70%), “cell cycle” (i.e., ≈78%) and “cell death” (i.e., ≈74%) processes (Supplementary Material 3), mirroring the pattern of MGO-perturbed genes. Furthermore, the analysis of protein-protein interactions (PPI) revealed that most of the proteins - whose expression is perturbed upon MGO exposure and whose gene promoters are bound by NRSF/REST - are interconnected and belong to cell processes that RNA-Seq identified as altered by MGO (Figure 4C; Supplementary Figures S3A–C). This finding further suggests a relevant role for NRSF/REST in these processes (Figure 4C; Supplementary Figure S3), mostly associated with the regulation of gene expression (Supplementary Material 3). Focusing on the most significant and robust PPI networks for NRSF/REST (Figure 4C), we disclosed multiple factors previously described as responsible for, or contributing to, the progression of DR and other retinal diseases, or associated with retina formation and homeostasis (Supplementary Material 3). Noteworthy, ≈54% of all DEGs - labeled “DR-related” in the Human Gene Database GeneCards (Supplementary Material 1) - are *bona fide* targets of NRSF/REST (i.e. 199 genes; Supplementary Material 3). Considering the concordance analysis between MGO-induced transcriptional changes and independent diabetic endothelial datasets (Supplementary Figure S1C), the predominance of concordantly regulated genes includes several NRSF/REST-related genes (Supplementary Figure S4A), supporting conservation of NRSF/REST-dependent transcriptional remodeling induced by MGO across independent models of diabetic endothelial dysfunction. Consistently, NRSF/REST-bound DEGs in hRECs (Figure 4B; Supplementary Material 3) significantly overlapped with genes differentially expressed in independent transcriptomic datasets from diabetic retinal microvascular endothelial cells and PDR samples (Lam et al., 2017; Maurissen et al., 2024) (Supplementary Figure S4B), reinforcing the robustness of the identified transcriptional signature. Moreover, we also disclosed that proteins interacting with NRSF/REST-associated PPI networks contain binding sites for MGO-perturbed miRNAs. Particularly, two (i.e., miR-92a-3p and miR-26b-5p) out of the seven selected miRNAs - identified as targeting the core subset of MGO-perturbed genes (Figure 3C) - contain binding sites in REST transcript (Supplementary Material 2). Taken together, these findings further suggest the role of NRSF/REST as a key node within the transcriptional network perturbed by MGO in hRECs.

**FIGURE 4:**
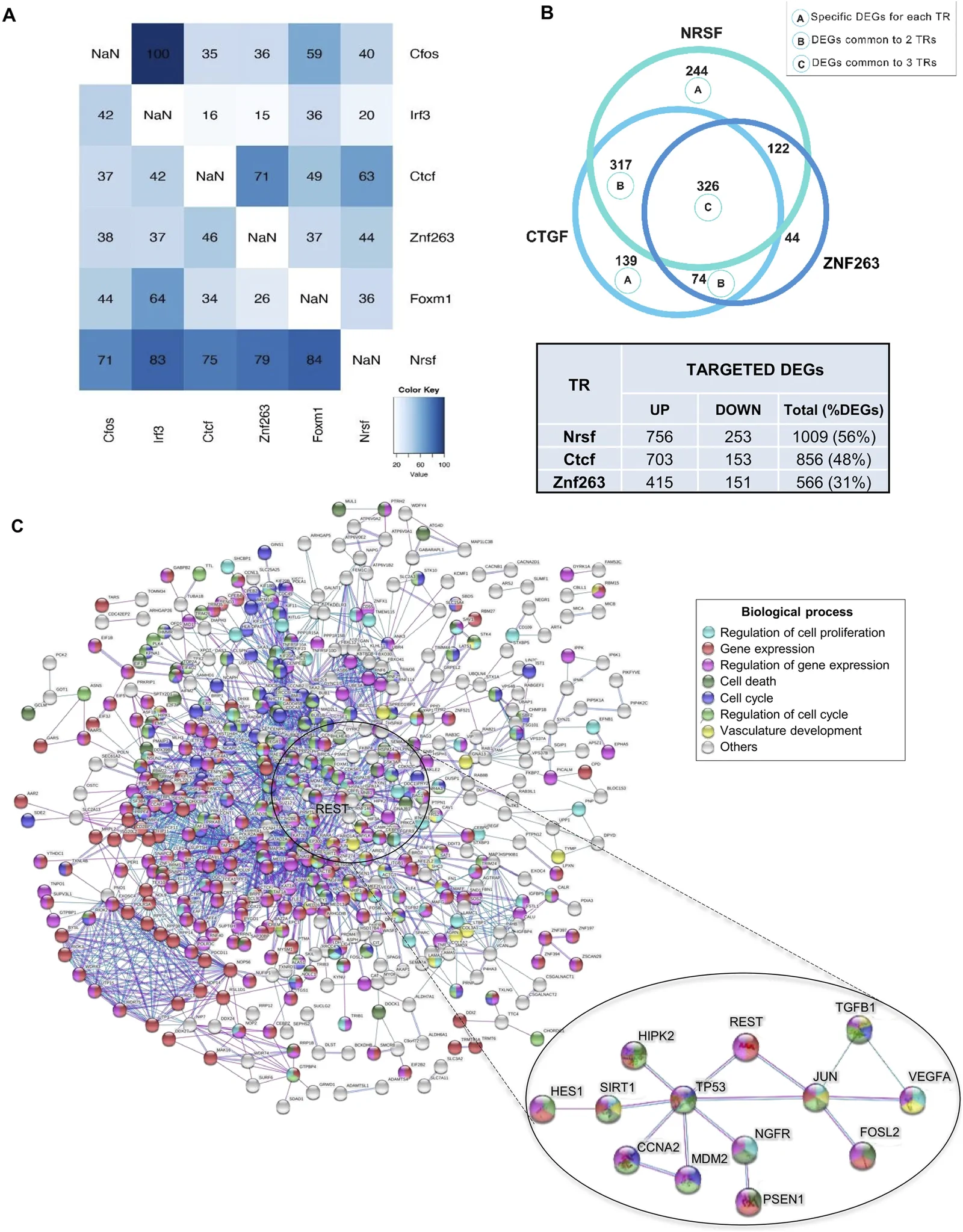
Identification of NRSF/REST as a candidate key transcriptional regulator of the MGO-responsive transcriptional network. **(A)** Target overlap matrix indicates the overlap between target genes of a subset of transcriptional regulators selected as enriched for binding sites in DEGs, according to ENCODE ChiP-Seq data. Each column reports transcription factors with the percentage of target genes common to factors indicated on each row. **(B)** Table showing the number of DEGs targeted by the three selected transcription factors (right panel) and Venny diagram displays the overlap between these subsets (left panel). **(C)** Graphical representation of the high-confidence Protein-Protein Interaction (PPI) networks of DEGs targeted by NRSF/REST, analyzed by STRING tool (confidence score >0.7). Protein interactions are shown as blue lines for evidence from curated databases, whereas pink lines indicate experimentally validated interactions. The different colors indicate the involvement of each protein to the selected biological processes (left panel). The map defines a core cluster of the NRSF/REST PPI network (right panel).

### 3.4 NRSF/REST mediates the molecular and functional effects of MGO in hRECs

To validate the hypothesis that the alteration of REST may be an early event following MGO exposure, we treated hRECs for 24 and 72 h with sublethal doses of MGO. As reported in Figure 5A, a more pronounced effect on REST over-expression was evident at 24 h, whereas a milder increase was measured at 72 h (according to RNA-Seq data, Supplementary Material 1). Moreover, we excluded any potential confounding effect possibly induced by FBS or of the endothelial cell growth supplement – contained in cell medium – evaluating NRSF/REST in presence and absence of these factors (Supplementary Figure S4C). Additionally, considering the curated PPI network of NRSF/REST (Figure 4C), we selected *TP53* and *FOSL2* genes as putative downstream mediators of NRSF/REST. Notably, in line with ENCODE ChIP-Seq data (Supplementary Material 3) and the presence of NRSF/REST binding sites within the putative promoters of *TP53* and *FOSL2* genes across multiple cell types (Supplementary Figure S4D), the knockdown of REST mRNA by specific siRNAs (Supplementary Figure S4E) reduces the expression levels of these genes (Figure 5B). Conversely, hRECs exposed to MGO for 24 h display a strong upregulation - not only of NRSF/REST - of these genes (Figure 5C). Thus, we assessed the effects of *REST* silencing on functionality of hRECs exposed to MGO. Notably, *REST* knockdown is sufficient to improve the deficit of hREC migration measured upon MGO exposure (Figure 6A), also inducing a significant recovery of tube formation capacity, in terms of number of nodes and loops (Figure 6B). Consistently, in a co-culture system used to assess endothelial–pericyte crosstalk, the silencing of *REST* in hRECs enhances the recruitment of hPCs impaired by MGO exposure (Figure 6C).

**FIGURE 5:**
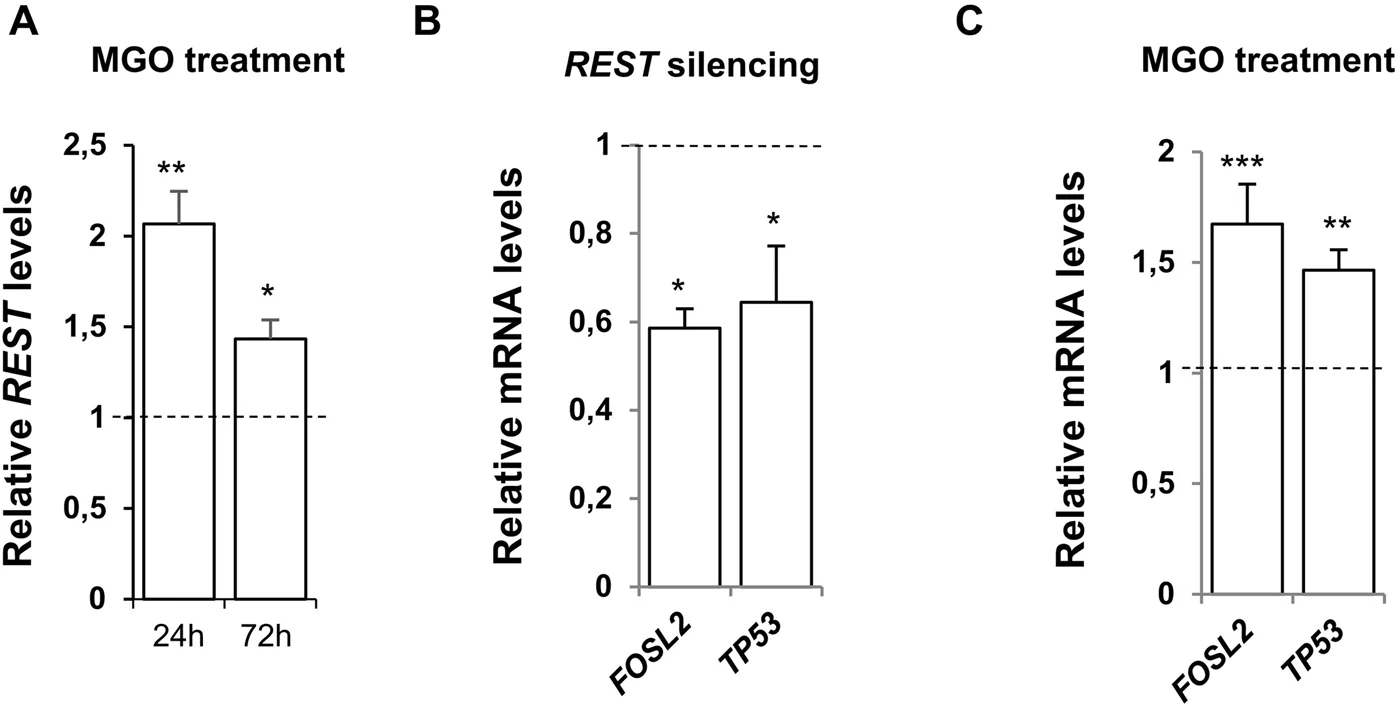
MGO modulates REST expression and related genes in hRECs. **(A)** Relative mRNA quantification (qPCR) of *REST* expression in hRECs treated with MGO 500 μM for 24 or 72 h. Data are reported as mean ± SEM vs. untreated control cells (dotted line) of independent experiments (n = 5). *PPIA* was used as reference. Comparison between groups was performed using Student’s t-test, with *p ≤ 0.05; **p ≤ 0.01. **(B,C)** Relative mRNA quantification (qPCR) of *FOSL2* and *TP53* expression in hRECs transfected with *REST* siRNAs **(B)** or treated with MGO 500 μM for 24 h **(C)**. Data are reported as mean ± SEM vs. control cells - i.e., transfected with scrambled siRNAs in **(B)** or treated with Veh **(C)**; dotted line - of independent experiments (n = 5). PPIA was used as reference gene. Comparison between groups was performed using Student’s t-test, with *p ≤ 0.05; **p ≤ 0.01; ***p ≤ 0.001.

**FIGURE 6:**
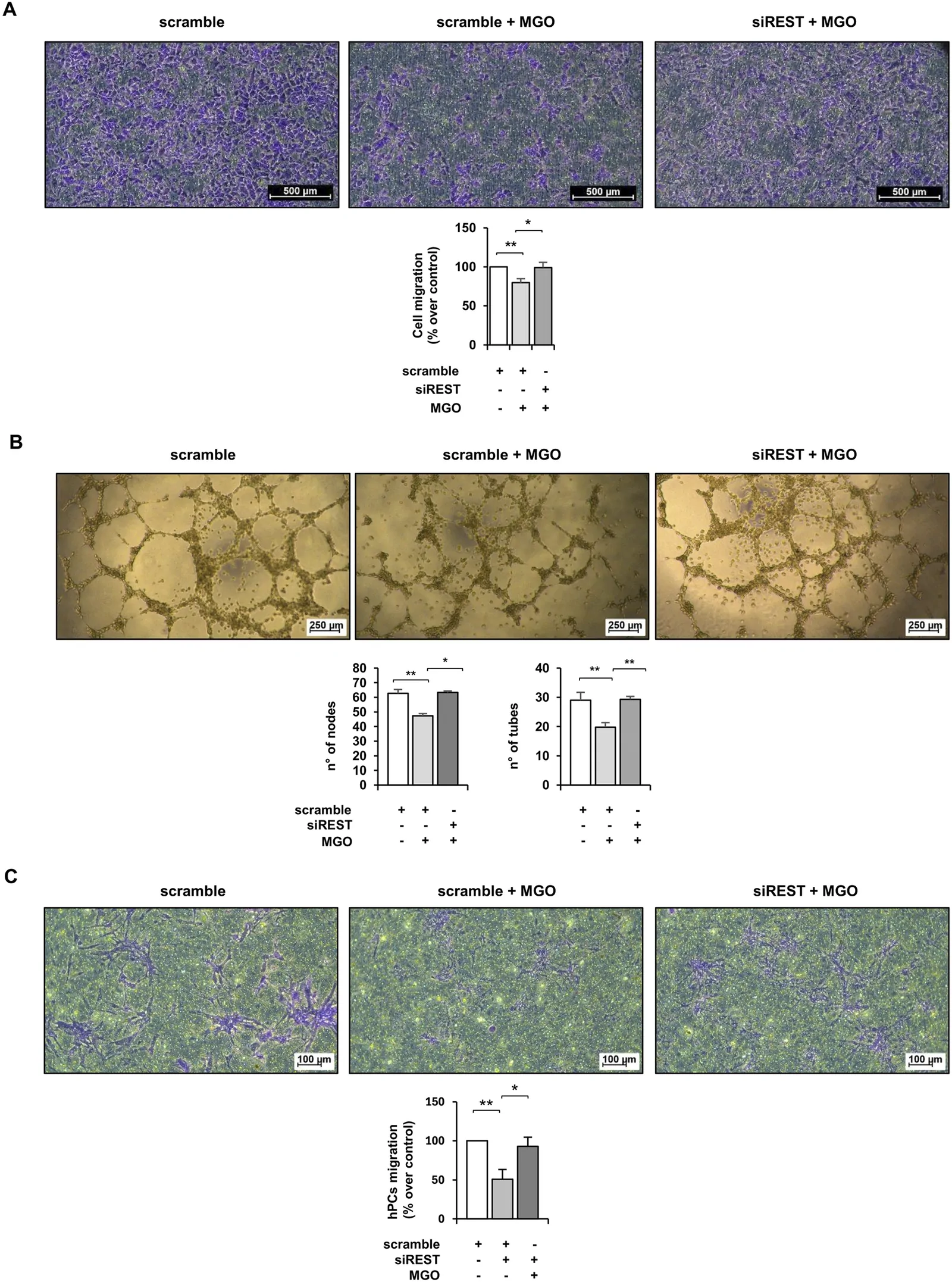
*REST* silencing counteract MGO-induced endothelial dysfunctions. **(A)** Representative optical microscopy images (left panel) at ×5 magnification (scale bar: 500 μm) of migrated hRECs treated or not with MGO 500 μM for 72 h and transfected with non-targeting siRNA (scramble and scramble + MGO) or with *REST* siRNAs (siREST + MGO). Cells were exposed to FBS as pro-angiogenic stimulus and stained with crystal violet after 24 h. Bar graph (right panel) indicates the migrated hRECs number, reported as mean ± SEM of independent experiments (n = 8). *p ≤ 0.05. **p ≤ 0.01. **(B)** Representative optical microscopy images at ×5 magnification (scale bar: 250 μm) of hRECs treated or not with MGO 500 μM for 72 h and transfected with non-targeting siRNA control (scramble and scramble + MGO) or with *REST* siRNAs (siREST + MGO), seeded on Matrigel-coated 96-well plate and analyzed after 6 h. Bar graphs indicate the number of nodes (left panel) and tubes (right panel), analyzed and quantified by ImageJ. Data are reported as mean ± SEM of independent experiments (n = 6). **(C)** Representative optical microscopy images at ×10 magnification (scale bar: 100 μm) of migrated hPCs in response to co-culture with hRECs, treated or not with MGO and transfected with non-targeting siRNA control (scramble and scramble + MGO) or with *REST* siRNAs (siREST + MGO), and stained with crystal violet after 24 h. Bar graph indicates the mean ± SEM of the number of migrated hPCs in independent experiments (n = 6), expressed as % over control (scramble). Comparison between groups was performed using Student’s t-test, with *p ≤ 0.05 and **p ≤ 0.01.

Overall, these findings demonstrate that NRSF/REST is a promising candidate contributing to both transcriptional and functional MGO-induced alterations in hRECs.

## 4 Discussion

Chronic hyperglycemia is the primary determinant of macular edema and proliferative retinopathy, key sight-threatening endpoints in DR (Wong et al., 2016; Liu et al., 2025). The cascade of events initiated by oxidative stress sustains the inflammatory environment perpetuating vascular damage (Brucklacher et al., 2008; Capitão and Soares, 2016; Tang et al., 2024; Liu et al., 2023). The compromission of blood-retinal barrier integrity is attributed to pericyte loss and endothelial cell dysfunction, causing a defective crosstalk within the neurovascular unit (Hammes, 2018; Saxena et al., 2025). Recent studies have emphasized the pivotal role of MGO in DR (Günthner et al., 2019; Klochkov et al., 2024; Wen et al., 2026; Ramasamy et al., 2006). The evidence that the association between increased serum levels of the specific MGO-derived hydroimidazolone and retinopathy in T2DM patients is not dependent of hitherto known associated factors, such as hemoglobin A1c (Fosmark et al., 2006), suggests the notion that MGO *per se* acts as a driving cause of retinal damage. Nonetheless, the specific molecular mechanisms accounting for the retinal damage remain elusive. Moreover, the advancement in understanding the molecular basis of DR is strongly limited by challenges in human models and the paucity of comprehensive studies. Our analysis, based on the use of primary hRECs, shows the strong impact that MGO exerts on these cells, shedding light on relevant molecular mechanisms underlying DR etiology. This study systematically investigates the effects of MGO exposure on glycation adduct formation, on functional features (e.g., the angiogenic capacity) as well as on transcriptome and miRNome of endothelial cells. The observed increase in protein glycation adducts highlights how investigating MGO-effects in hRECs is a relevant aspect to understand its role on endothelial damage in DR. Importantly, MGO exerts its biological effects primarily through the formation of covalent adducts with proteins. Therefore, the experimental condition selected for this study was not intended to reproduce a specific circulating or tissue concentration of free MGO on a one-to-one basis. Rather, it was chosen as a model of sustained dicarbonyl stress in hRECs. In this context, the extent of MGO-conjugated protein accumulation represents a more stable and functionally relevant readout of MGO-induced damage. Notably, the 65% increase of MGO-conjugated proteins observed in our experimental model is consistent with the *in vivo* accumulation of MGO-derived hydroimidazolone (MG-H1) reported in the serum of patients with T2DM, which showed a 58% increase compared with non-diabetic controls (Kilhovd et al., 2003). More specifically, it is also consistent with the accumulation of dicarbonyl modified proteins reported in the vitreous of patients with DR, showing 60%–80% increase compared with control subjects (Choudhuri et al., 2013; Fosmark et al., 2007). Moreover, vitreous levels were reported by the authors to correlate with serum levels. Overall, these clinical evidence supports the pathophysiological relevance of our experimental model.

Functionally, hRECs exposed to MGO exhibited impaired migratory ability, as well as reduced capacity to form tube-like structures and to recruit pericytes, indicating a detrimental impact of MGO on the angiogenic capacity of these cells and suggesting a link between MGO-induced alterations and DR pathogenesis.

Furthermore, our work indicates an extended perturbation of both cell transcriptome and miRNome induced by MGO, resulting in a global alteration of expression levels of multiple genes involved in cell cycle, cell death, proliferation, vasculature development and adhesion. Noteworthy, most of the MGO-perturbed genes encode for transcriptional modulators, either promoting or inhibiting gene transcription. Interestingly, a notable portion of genes - whose expression is altered upon MGO exposure - can be classified as DR-related in GO and pathway analysis, strengthening the relevance of our results on this primary *in vitro* model. Further enforcing the evidence that MGO has a strong impact on gene expression regulation, our results show a wide perturbation of the miRNA network upon MGO exposure, identifying also key miRNAs associated with DR-related processes. Through an exploratory prediction-based integrative analysis of transcriptomic and miRNome data, we identified a core subset of MGO-deregulated mRNAs and miRNAs, revealing a highly interconnected mRNA–miRNA regulatory network and coordinated perturbation of common biological processes following MGO exposure. Moreover, using a hypothesis-generating approach to select candidate transcriptional regulators, we identified NRSF/REST as a major candidate of the wide transcriptional deregulation following MGO exposure. Indeed, the observation that a large proportion of MGO-deregulated genes are enriched for NRSF/REST binding sites in public ENCODE ChIP-Seq datasets suggests a potential involvement of this factor in the endothelial dysfunction, supporting its role as a candidate mediator of the MGO-mediated effects. Notably, Yao et al. (Yao et al., 2007) reported that high levels of MGO, in mouse kidney endothelial cells, lead to modifications in mSin3A- a cofactor of NRSF/REST - compromising in turn the complex and increasing Ang-2 expression. Interestingly, our results indicate a marked increase of *REST* expression levels as an early event following MGO exposure, suggesting it may work as a possible trigger for the activation of a downstream cascade ultimately leading to several MGO-related detrimental effects. Moreover, the integration of transcriptome and miRNome analyses highlighted a possible contribution of miR-92a-3p and miR-26b-5p, downregulated by MGO, on NRSF/REST expression. The computational prioritization of NRSF/REST as a candidate regulator is further supported by the concordance with independent diabetic endothelial transcriptomic datasets, showing a significant overlap between NRSF/REST-bound DEGs identified in hRECs and genes differentially expressed in diabetic retinal microvascular endothelial cells and PDR samples (Lam et al., 2017; Maurissen et al., 2024). Moreover, our exploratory integrative analysis using public ENCODE ChIP-seq datasets identified putative NRSF/REST binding sites at the promoters of several MGO-responsive, DR-related genes, including *TP53* and *FOSL2*, rapidly induced after 24 h of MGO exposure. Although these findings do not demonstrate direct promoter occupancy in our experimental model, they provided a strong rationale for prioritizing functional perturbation approaches through dedicated *REST* knockdown experiments. Accordingly, *REST* silencing experiments demonstrated a marked reduction of *TP53* and *FOSL2* expression in knockdown cells. Beyond the well-established role in cell proliferation and differentiation, Fosl2 induction has been reported as a consequence of axonal injury in retinal ganglion cells (Fernandes et al., 2013), whereas the low-grade upregulation of Tp53 inhibits endothelial cell migration (Panta et al., 2017; Leone et al., 2023). Moreover, the ability of *REST* silencing to counteract the detrimental effects of MGO on migration, tube-forming capacity and recruitment of pericytes in hRECs, provides strong functional evidence that NRSF/REST contributes to endothelial dysfunction induced by MGO in the context of DR. These findings extend the transcriptomic predictions by demonstrating that NRSF/REST is not only associated with MGO-induced transcriptional remodeling but also functionally involved in the impaired angiogenic phenotype of hRECs.

Despite these findings, the present study does not fully reproduce the multicellular architecture and dynamic signaling of the retinal neurovascular unit. In addition, because the primary hRECs used in this study were obtained from a single healthy donor, donor-to-donor biological variability could not be assessed. Although the experiments were repeated using independent vials from different cell batches, future studies using hRECs derived from multiple donors, including diabetic and non-diabetic subjects, will be required to further validate the generalizability of these findings. Nevertheless, this reductionist approach represents a major strength for defining direct, cell-autonomous molecular responses to MGO, while minimizing the confounding contribution of multiple diabetes-associated factors that coexist *in vivo*. In this context, our study provides valuable insight into the molecular mechanisms triggered by MGO in hRECs, identifying NRSF/REST as a promising mediator of MGO-induced endothelial dysfunction. These findings provide a mechanistic framework that should now be validated in more physiologically relevant models, including retinal organoids, multicellular co-culture systems and *in vivo* models of DR. Overall, our results pave the way for future mechanistic and translational studies aimed at identifying therapeutic targets and developing intervention strategies to mitigate the detrimental impact of MGO on retinal endothelial cells and, ultimately, on DR progression.

## Data Availability

The datasets presented in this study can be found in online repositories. The names of the repository/repositories and accession number(s) can be found below: https://www.ncbi.nlm.nih.gov/geo/, GSE263470 https://www.ncbi.nlm.nih.gov/geo/, GSE263471.
