# Supplementary material for: Methylglyoxal hampers the angiogenic capacity of human retinal endothelial cells modifying cell transcriptome with the contribution of NRSF/REST
Source: Front Cell Dev Biol. 2026 Oct 2;14:1762167. doi: 10.3389/fcell.2026.1762167 (PMC13672637; doi:10.3389/fcell.2026.1762167)
Supplement: Supplementary file 1 [file DataSheet1.pdf]

Supplementary Figure 1

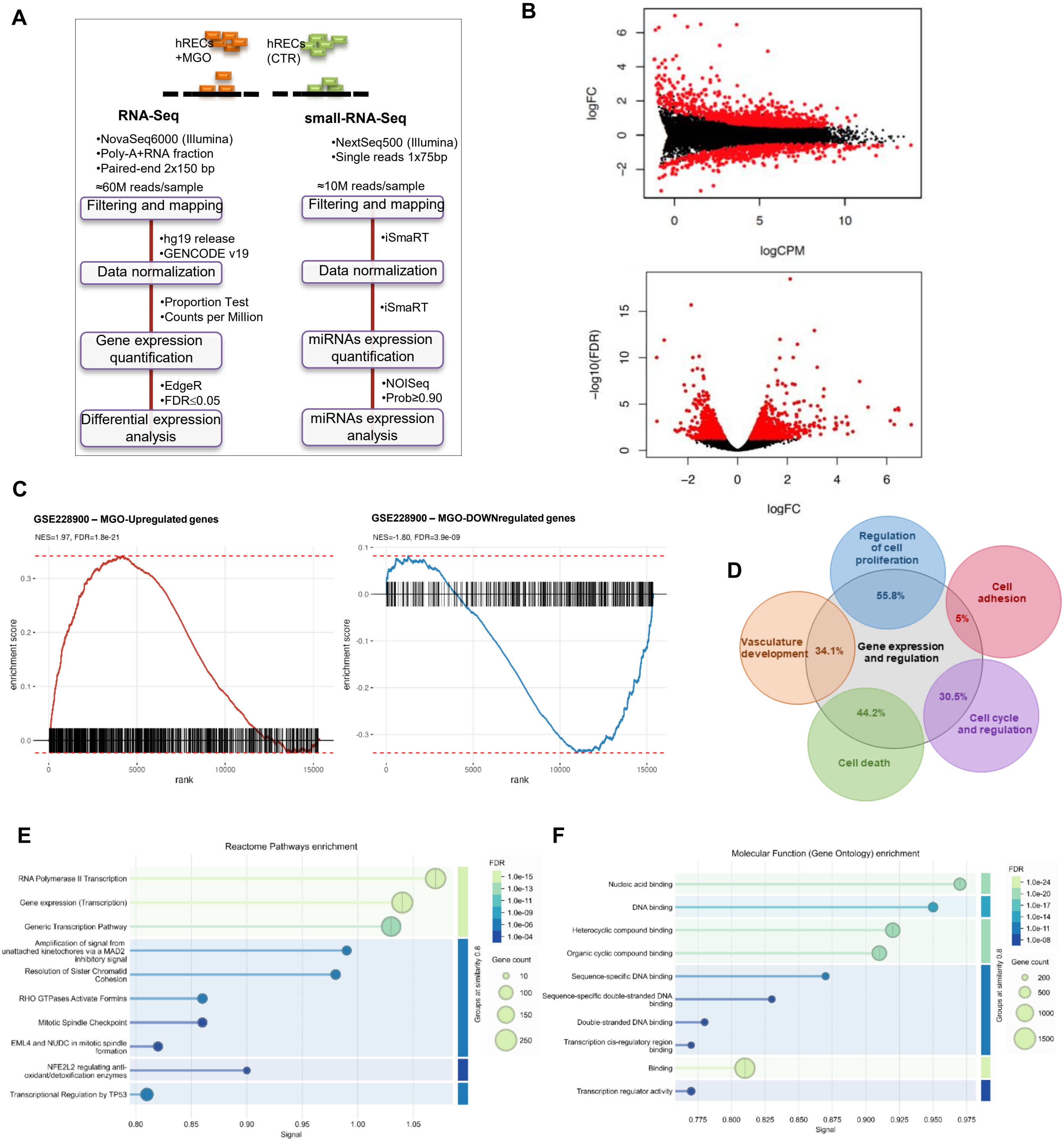

Figure S1. Workflow of NGS experiments and analysis of differentially expressed genes (DEGs). (A) Protocols and workflows for RNA-Seq (left side) and smallRNA-Seq (right side) data generation and analysis. **(B)** MA plot (right panel) and Volcano plot (left panel) of DEGs. **(C)** Gene set enrichment analysis (GSEA) of MGO-regulated genes (DEGs) queried against ranked gene lists from diabetic primary human retinal microvascular cells (GSE228900, upregulated-gene signature NES=1.97, FDR=1.8e-21; downregulated-gene signature NES=-1.80, FDR=3.9e-09), indicating concordance between MGO-induced transcriptional programs and diabetic endothelial state. **(D)** Venn diagram showing the proportion (%) of DEGs involved in distinct biological processes with functions in gene expression and regulation. **(E)** STRING analysis (based on Reactome database) of pathways enriched and **(F)** molecular functions of DEGs, with relative gene counts and FDR.

Supplementary Figure 2

CELLULAR COMPONENTS

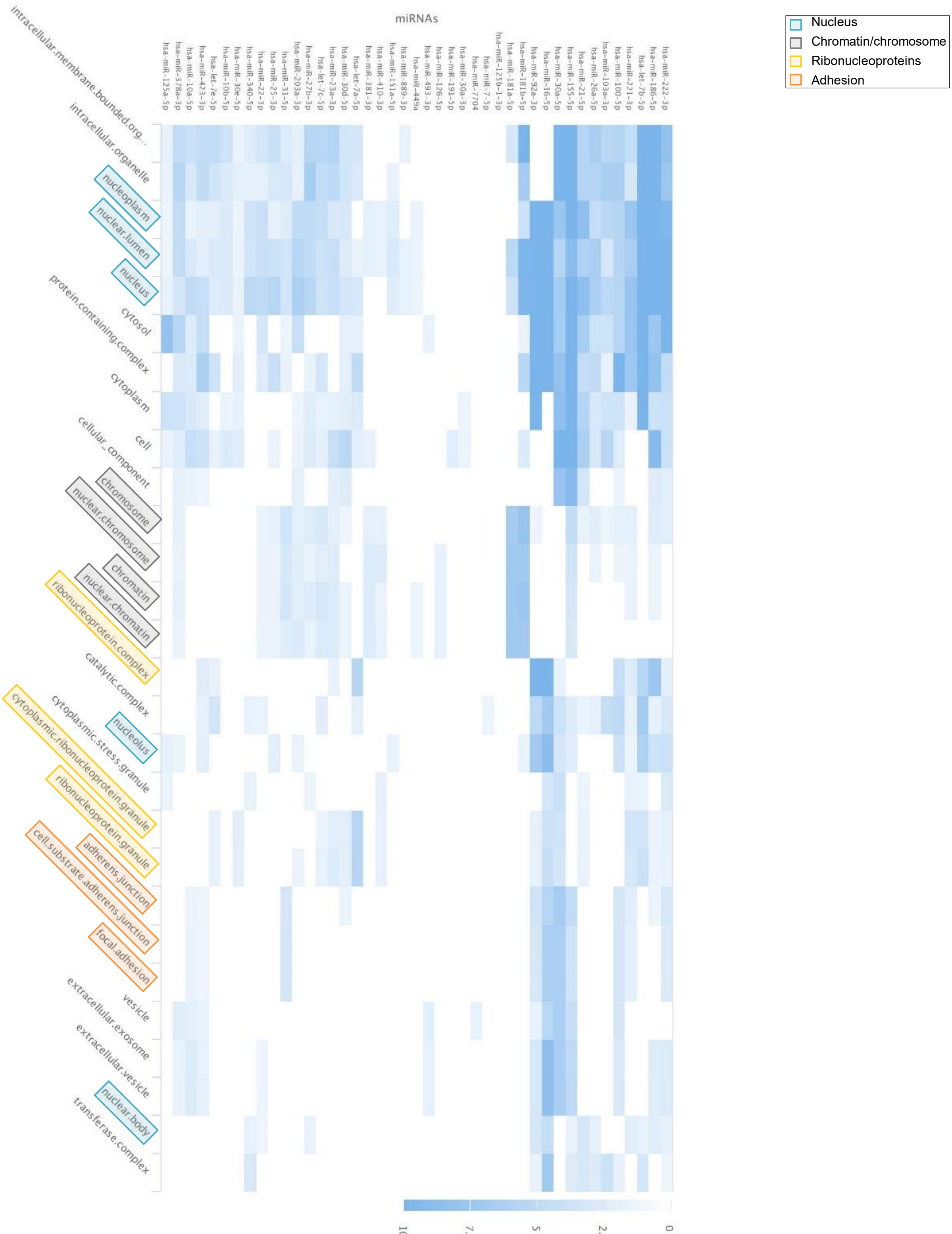

Figure S2. Cellular components of genes regulated by altered miRNAs. (A) Heatmap generated in miRPathDB 2.0 of cellular components of genes regulated by altered miRNAs, according to the KEGG database. Different colors indicate the main pathways/cellular components including the specific signaling/components.

Supplementary Figure 3

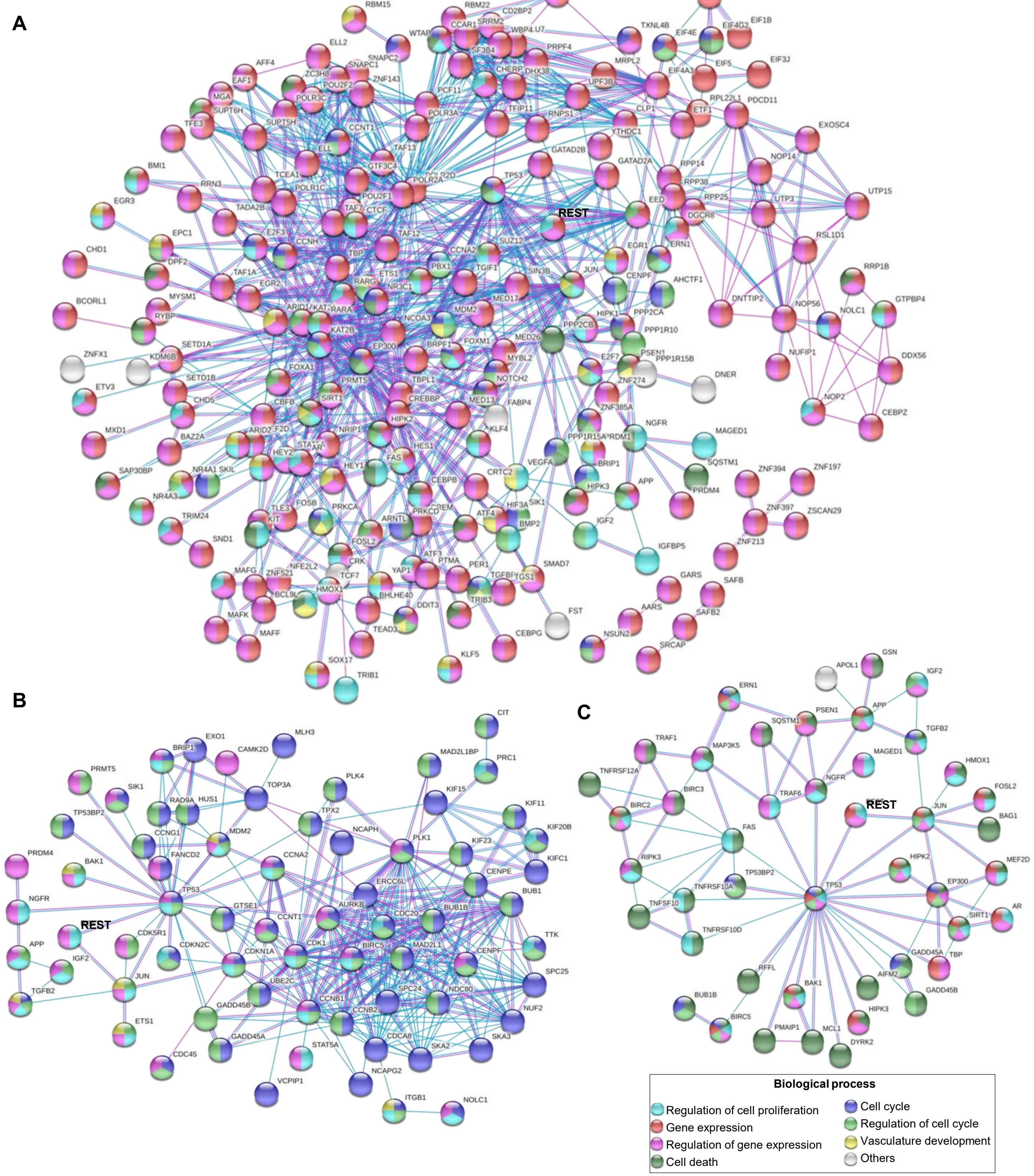

Figure S3. Analysis of Protein-Protein Interaction (PPI) networks of genes altered by MGO and targeted by NRSF/REST. (A) Graphical representation of the high-confidence Protein-Protein Interaction (PPI) networks - analyzed by STRING tool (confidence score > 0.7) - of DEGs targeted by NRSF/REST and involved in gene expression and regulation, **(B)** cell cycle and regulation and **(C)** cell death, according to DAVID Gene Functional Classification Tool. Protein interactions are shown as blue lines for evidence from curated databases, whereas pink lines indicate experimentally validated interactions. The different colors indicate the involvement of each protein to the selected biological processes.

Supplementary Figure 4

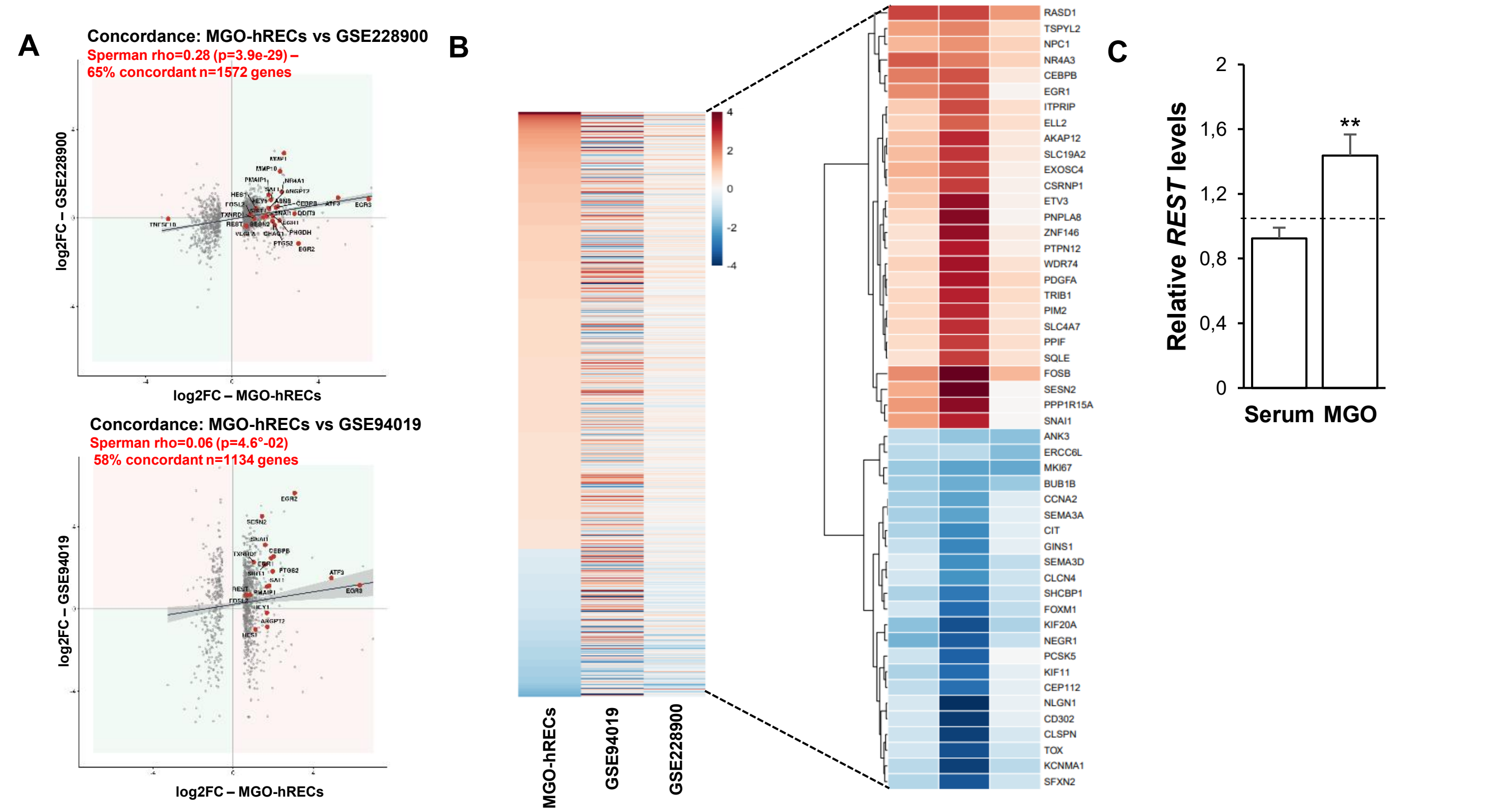

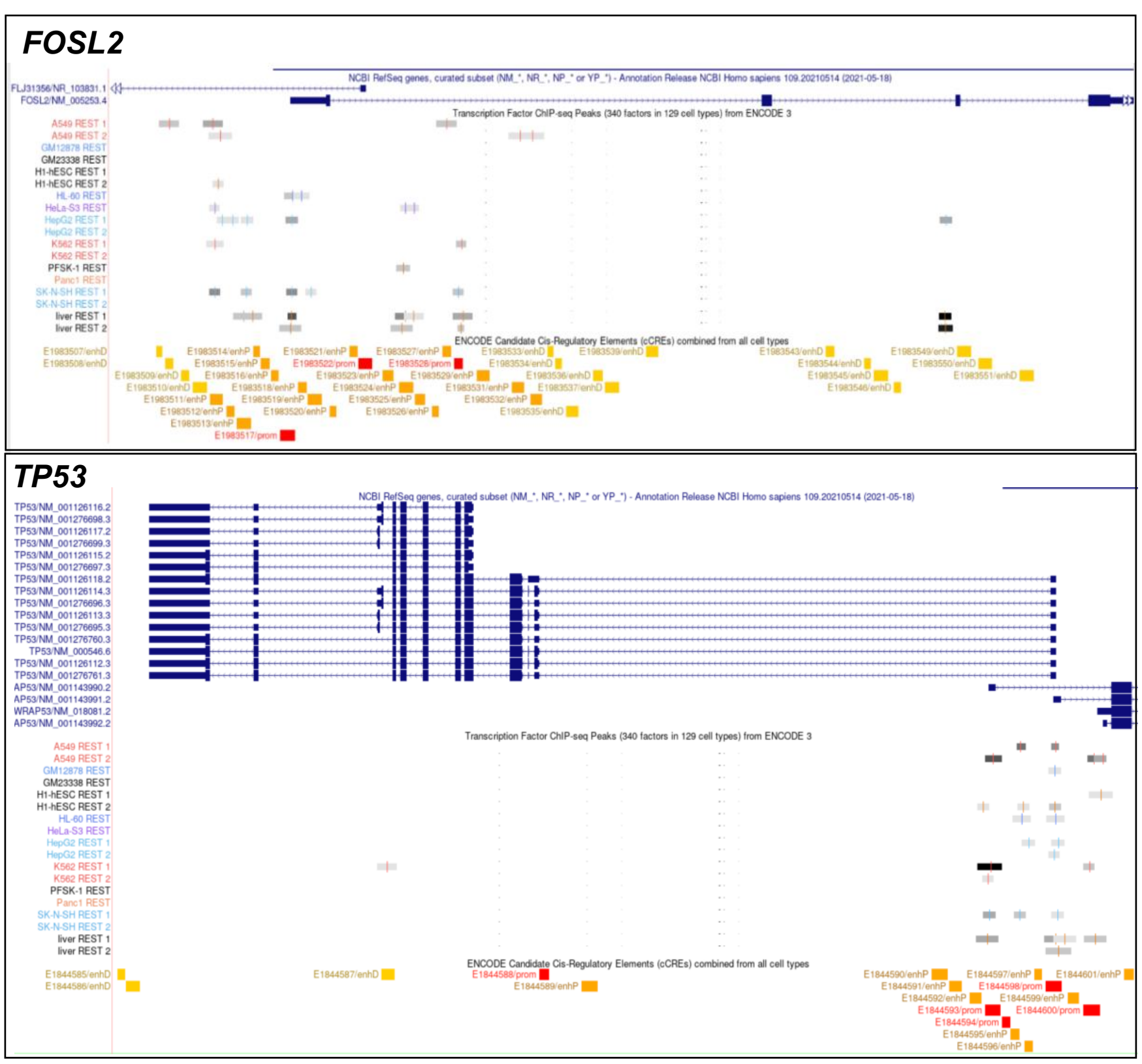

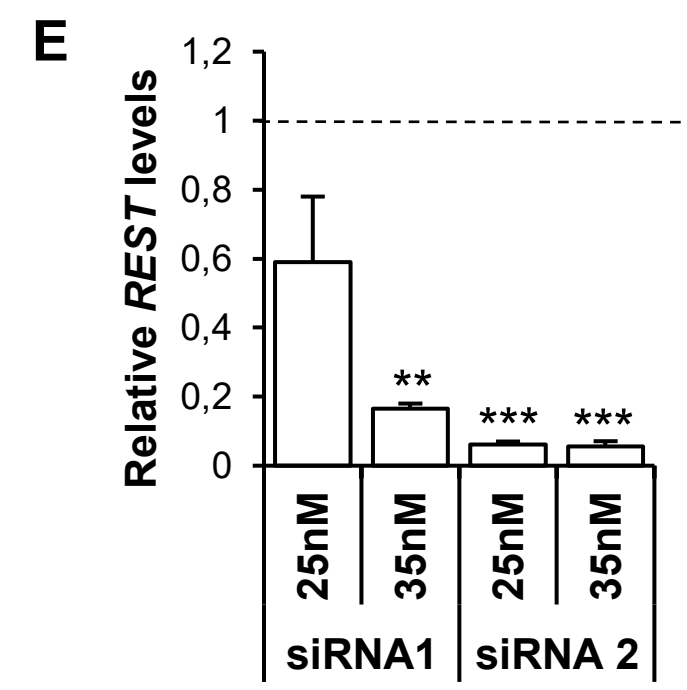

Figure S4. Expression, silencing and binding of NRSF/REST. (A) Scatter plots comparing log2 fold changes obtained in MGO-treated hRECs with those from the GSE228900 (top) and GSE94019 (bottom) datasets. Each point represents a shared gene; red labels indicate selected significantly deregulated genes. Shaded quadrants denote concordant (upper-right and lower-left) or discordant (upper-left and lower-right) regulation. Spearman correlation coefficients (ρ), associated P values, and the percentage of concordantly regulated genes are reported for each comparison.
(B) Hierarchical clustering heatmap illustrating the transcriptional concordance between MGO-treated hRECs and independent datasets (GSE94019 and GSE228900; concordance scores computed over all REST-bound DEG: GSE94019 = 61% concordant - Spearman rho=0.08; GSE228900 = 65% concordant - Spearman rho=0.30). A dendrogram highlights clustering of gene sets with conserved directional regulation across datasets. A zoomed-in panel shows the top 50 REST-bound DEGs with the strongest concordance. (**C**) Relative mRNA quantification (qPCR) of *REST* expression in hRECs exposed to serum or MGO without serum. Data are reported as mean ±SEM *vs* control cells (Veh without serum; dotted line) of four independent experiments. *PPIA* was used as reference. **p val ≤ 0.01. **(D)** *TP53* and *FOSL2* genomic regions view showing multiple *REST* binding sites in promoter regions (Genomic UCSC genome browser). **(E)** Relative mRNA quantification (qPCR) of *REST* expression in hRECs transfected with two different *REST* siRNAs. Data are reported as mean ±SEM *vs* control cells (scrambled siRNAs; dotted line) of four independent experiments. *PPIA* was used as reference. **p val ≤ 0.01, ***p val ≤ 0.001.

Table S1. Primer sequences of genes analyzed by qPCR

Gene	Sense primer (5'-3')	Antisense primer (3'-5')	Melting Temperature (°C)	PCR product (bp)
PPIA	TACGGGTCCTGGCATCTTGT	GGTGATCTTCTTGCTGGTCT	60	196
REST	GCTGCGGCTACAATACTAATC	GAGTAAGGACAAAGTTCACAT	60	277
FOSL2	CATCACGACCAGCCAGGAC	AGCCAGCTTGTTCCTCTCC	60	253
TP53	CCCCTCCTCAGCATCTTATC	GTGTGATGATGGTGAGGATG	60	204
